# Supplementary material for: A Laccase Gene Reporting System That Enables Genetic Manipulations in a Brown Rot Wood Decomposer Fungus Gloeophyllum trabeum
Source: Microbiol Spectr. 2023 Jan 18;11(1):e04246-22. doi: 10.1128/spectrum.04246-22 (PMC9927100; doi:10.1128/spectrum.04246-22)
Supplement: Supplemental file 1 — Supplemental material. Download spectrum.04246-22-s0001.pdf, PDF file, 0.7 MB [file spectrum.04246-22-s0001.pdf]

Supplementary Figures:

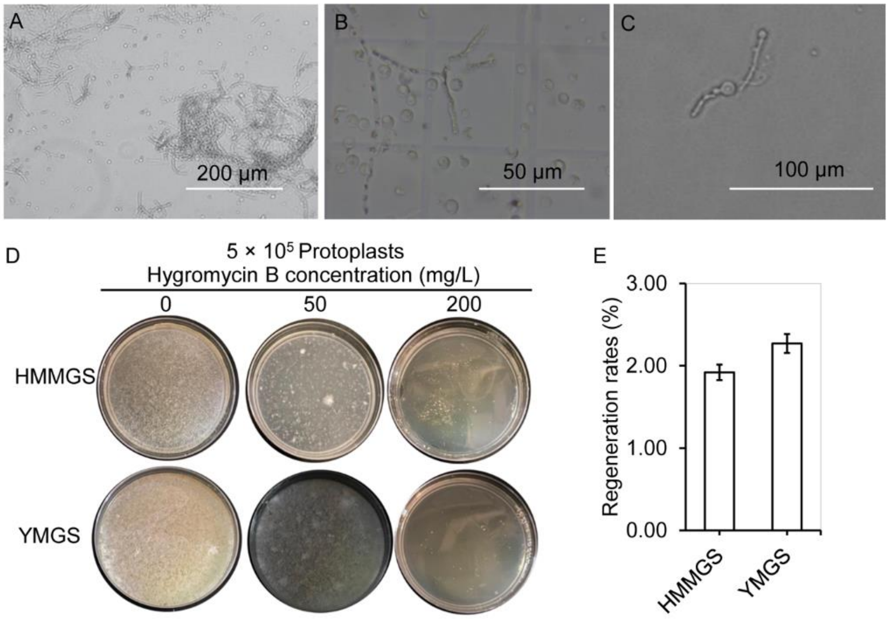

Fig. S1. Regeneration tests of *G. trabeum* protoplasts. (A and B) *G. trabeum* protoplast cells generated after 6 hours of lysing treatment. (C) Germination of protoplasts in YMGA solid media. Scale bars were shown in the images. (D) Hygromycin sensitivity test with $5x10^6$ protoplasts were mixed with HMMG or YMGA solid media containing 0.5 M sucrose for regeneration. (E) The regeneration rates of protoplasts on HMMG and YMGA media.

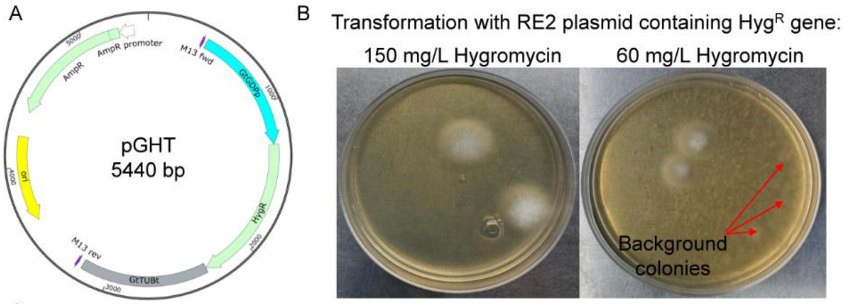

C

Experiments	DNA (μg)	Protoplasts	HygB-resistant transformants	PCR positivity	Transformation efficiency*
1	5	10^7	235	3/8 (38%)	17.86
2	8	$2×10^6$	50	7/13 (54%)	16.875
3	10	$2×10^7$	410	10/15 (67%)	13.735

*Transformants per μg plasmid per 10^7 protoplasts

Fig. S2. Transformation of pGHT plasmid into *G. trabeum*. (A) Map of the pGHT vector containing the hygromycin-resistant gene (Hyg^R). (B) Comparison of two hygromycin concentrations used in overlaying media for transformation. Both 60 mg/L and 150 mg/L provided successful selection of hygromycin-resistant colonies, but a lot of background colonies emerged on the first layer media when overlaid with 60 mg/L hygromycin. (C) The summary table for three independent pGHT transformation experiments.

Fig. S3. Paired correlation analyses among enzyme activity, mRNA level, and ABTS halo in the laccase transformants. Laccase enzyme activities and mRNA levels were measured in HMM-Glucose media (as in Fig. 2E), and the ABTS halo sizes were measured by ABTS plate assay (as in Fig. 2B). Polynomial or linear regression models were used. (D) Correlation matrix of the laccase-producing factors in transformants.

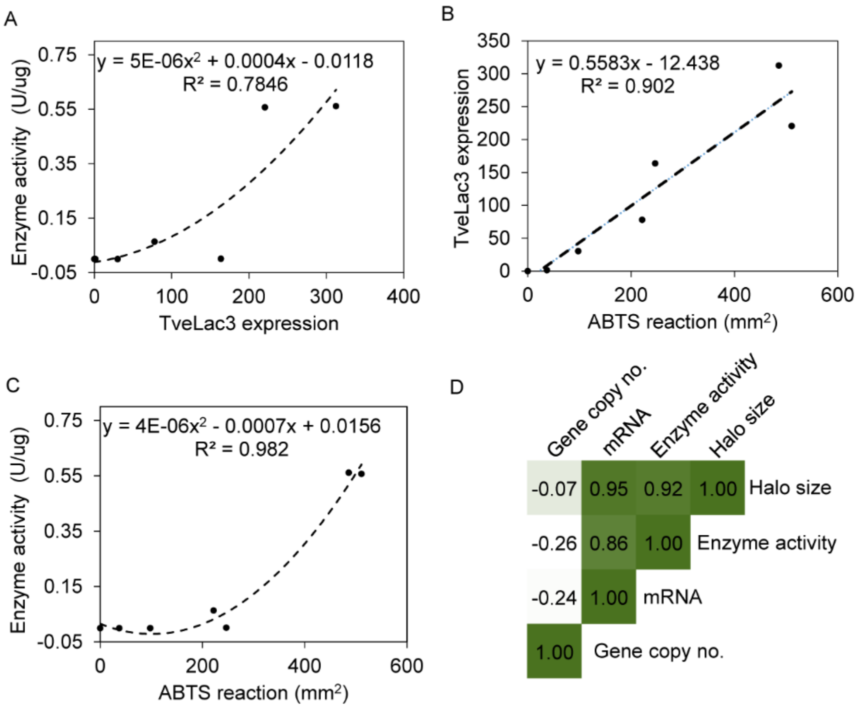

Table S1. Primers used for vector construction in this study.

Primers	Sequences (5' to 3')
GAPDH-HY-F1	TGCCTGCAGGCGGACTGAATCAAGGTGAGTG
GAPDH-HY-R1	TTCAGGCTTTTTCATGACTGGTGATGAAGAGGGAGAT
GAPDH-HY-F2	CTTCATCACCAGTCATGAAAAAGCCTGAACTCACCGC
GAPDH-HY-R2	CGAGAGACGCGCCTATTCCTTTGCCCTCGGACG
GAPDH-HY-F3	CAAAGGAATAGGCGCGTCTCTCGCT
GAPDH-HY-R3	TGCCTGCAGGCTCCTCAATCTGAGACTTCGCC
pGHT-OV-Lac3- F1	GTCTAAGAAACCATTCGGACTGAATCAAGGTGAGTGATG
pGHT-OV-Lac3- R1	GAAACCTCGACATGACTGGTGATGAAGAGGGAGA
pGHT-OV-Lac3- F2	TTCATCACCAGTCATGTCGAGGTTTCACTCTCTTCTCG
pGHT-OV-Lac3- R2	GAGAGACGCGCTTACTGGTCGCTCGGGTCG
pGHT-OV-Lac3- F3	CGACCAGTAAGCGCGTCTCTCGCTATGCC
pGHT-OV-Lac3- R3	CAACACCCGCTGACGCTCCTCAATCTGAGACTTCGCCG
Lac3-OV-pTub-F	TCGGTACCGCTTGAAGTATCAAACCGTTGCCA
Lac3-OV-pTub-R	CGCTGACTATAGTTTTCCAGCATGTCGAGGTTTCA
Lac3-OV- Lac3-F	TATAGTTTTCCAGCATGTCGAGGTTTCACTCTCTTCTC
Lac3-OV- Lac3-R	TCGACCCGAGCGACCAGTAAGGTGTTGAAA
Lac3-OV-tGA-F	GCGACCAGTAAGGTGTTGAAAGTGCGTGTGCAGA
Lac3-OV-tGA-R	ACTTGCGAGATGGCATCACATGAGTCTAGATCC
g Lac3-OV-pTub-F	TCGGTACCCGAGAGCTTGAATATGCGGTACAC
gLac3-OV-pTub-R	GACTGACGCGACACCTACAGCATGTCGAGGTTTCA
gLac3-OV-Lac3-F	CGACACCTACAGCATGTCGAGGTTTCACTCTCTTCTC
gLac3-OV-Lac3-R	TCGACCCGAGCGACCAGTAAATACAGACAT
gLac3-OV-tGA-F	CGACCAGTAAATACAGACATTTCCTTACCTCGGT
g Lac3-OV-tGA-R	GATGGCTGTCTGTGTGGTTACCTTTCTAGATCC
HphverifyF	GAGCCTGACCTATTGCATCTC
HphverifyR	CGAGTACTTCTACACAGCCATC
ppGpcrosspF	CTCCATCTTGTCCTCCTCATTC
TvLac3crosspR	GGTCATTCGGGTCGTAAACA
LacSouthernF	TTCGTCGTTGCTTCCCTTAC
LacSouthernR	TCGATGCTGAACGTGTAGTTG

Table S2. Primers used for RT-qPCR in this study.

Primers	Sequences	Amplification efficiency (%)
qTvLac3F1	CAACGAGGTCAACCTGCAC	87.78
qTvLac3R1	GCCGTTGATGAAGAAGTTGGT	
qGtPF6F	GTCTGAGAATGAGCTCGTCAA	96.91
qGtPF6R	TCACATTGCTCTTCGCTTCT	
qGteIF3F	CTTCCTTTGTCGAGCACCAT	88.86
qGteIF3R	TCCTTCTGTTTCTCGTTCGC	
qGTLac43770F	GTCGGCTCCCTTCAGATATT	96.09
qGTLac43770R	GGTCCAGTCTGCGATCTTAT	
qGTLac107459F	GGCGACTCACTCAACCATAAT	93.2
qGTLac107459R	AGGAATGATACTCGGTGTTTGG	
qGTLac127593F	ATTCTCGAGGCTAACCAGAAAG	98.2
qGTLac127593	CGCAAGATTGCTGCATTGAT	
qGTLac130426F	GAGGTGTAGACAAGCGATTCTT	90.87
qGTLac130426R	GGACAGGTAATTCAGGGTTCTG	

Table S3. Comparison of transformation efficiency in different wood-decay fungal species.[@]

Species	Protoplast (per ml)	Plasmid (μg)	Plasmid size (kb)	Promoter of marker gene	Transformation efficiency [#]	Reference
G. trabeum 15539	$0.2\text{-}2\times10^{7}$	5-10	5.4	GPD	13.7-17.9	This study
G. trabeum 15539	1×10^{7}	6-30	10	*Β*-tubulin, GPD	0.26-1 (4.5[*])	This study
Flammulina velutipes	1×10^{8}	10	7	GPD, TRP1	0.1-0.3	Maehara *et al*.[45]
Dichomitus squalens	$3\text{-}20\times10^{7}$	10	9	CaMV35S	0.25-2.6	Daly *et al*.[24]
Cerrena unicolor	2×10^{7}	250	14 to 17	CaMV 35S	0.104	Zhang *et al*.[46]

[@] All data were based on using hygromycin-resistant gene for transformation.

[*]The number of 4.5 indicates the potential outlier experiment in this study (as in Fig. 3C).

[#] Transformation efficiency is presented as transformant number per μg DNA per 10^{7} protoplasts.
